# Screening of Anti-Malarial Activity of Different Extracts Obtained from Three Species of *Scrophularia* Growing in Iran

**Published:** 2018

**Authors:** Fariba Heshmati Afshar, Abbas Delazar, Solmaz Asnaashari, Haleh Vaez, Elmira Zolali, Parina Asgharian

**Affiliations:** a *Drug Applied Research Center, Tabriz University of Medical Sciences, Tabriz, Iran. *; b *Department of Pharmacognosy, Faculty of Pharmacy, Tabriz University of Medical Sciences, Tabriz, Iran.*; c *Biotechnology Research Center, Tabriz University of Medical Sciences, Tabriz, Iran. *; d *Cardiovascular Research Center, Tabriz University of Medical Sciences, Tabriz, Iran.*; e *Student Research Committee, Faculty of Pharmacy, Tabriz University of Medical Sciences, Tabriz, Iran.*

**Keywords:** Anti-malaria, Scrophularia species, Cell free assay, Preliminary phytochemical analysis, GC-MS

## Abstract

*Scrophularia* genus belonging to the family of Scrophulariaceae, is a medicinal plant widely distributed in Iran. In the present study, the anti-malarial activity of different extracts of three Iranian endemic species of *Scrophularia* including *S. frigida*, *S. subaphylla* and *S. atropatana*, was screened by an *in-vitro* preliminary assay. The plant materials were extracted successively with n-hexane, dichloromethane (DCM), and methanol (MeOH) at room temperature by soxhlet extractor. In order to assess anti-malarial activity of obtained extracts, cell free β-hematin formation assay was applied. Amongst the extracts, DCM extract of *S. frigida* exhibited remarkable anti-malarial activity with IC_50 _value of 0.67 ± 0.11 mg/mL. In contrast, MeOH and n-hexane extracts of all plants illustrated insignificant or moderate activity in this assay. Furthermore, preliminary phytochemical analysis along with TLC and GC-MS analysis of potent extract (DCM extract of *S. frigida*) were performed for more clarification. These methods revealed that the notable anti-malarial activity might be due to the presence of active constituents like methoxylated flavonoids, methylated coumarins, and diterpenoids. From the nine extracts of different species of *Scrophularia*, DCM extract of *S. frigida* showed potent inhibitory activity on β-hematin formation assay. Hence, it seems that it is noteworthy to concentrate on purifying the active chemical constituents of DCM extract and determining the pure anti-malarial components.

## Introduction

**Figure 1:**
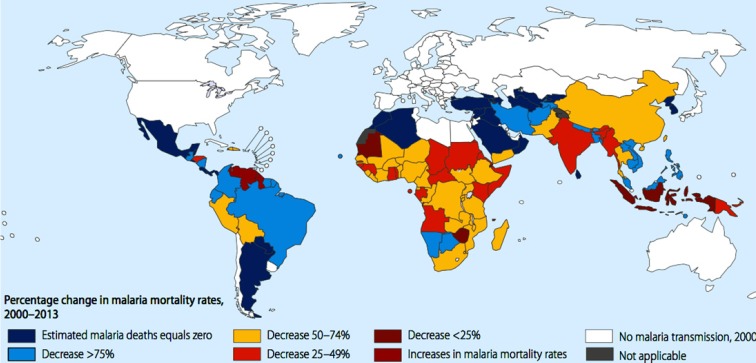
Percentage change in malaria mortality rates according to WHO estimation between 2000 to 2013.

Malaria is one of the common protozoal diseases which was widespread in many parts of the world especially in the tropical and sub-tropical zones and caused mortality and morbidity in patients (Figure 1) (1, 2). This global disease is caused by the bite of the Anophel mosquito which infected the host by the intracellular parasitic protozoa of plasmodium species (3, 4). Malaria as one of the oldest and fatal parasitic ailment in Iran is responsible for killing more than 60% cases of patients especially in southeastern area (5).

Hence, a substantial number of drugs has been considered for debating against this killer disease in recent years (6-8). As an illustration, chloroquine as one of the most inexpensive and routine anti-malarial medications was designated for treatment of malaria (9). However, the emergence of widespread chloroquine-resistant species in some regions as well as incapability of suppressing all stages of the parasite encouraged the researchers to make considerable attempt in discovering novel sources of plant derived drugs with highly clinical efficacy (10-12). Subsequently, artemisinin was one of the top lists of these herbal medicinal products with an endoperoxide sesquiterpene lactone structure, derived from the plant *Artemisia annua*. It exerts its potent anti-malarial effect by suppressing all stages of parasite in different ways including blocking the degradation of hemoglobin and inhibiting of toxic heme biocrystalization. (7, 13 and 14). Although artemisinin-based combination therapy has been represented by successful results in clinical treatments, the appearance of artemisinin-resistant parasites has inclined researchers for finding a new source of anti-parasitic drugs as the alternative therapies (15, 16). As a continuation of our screening program on biological activities of Iranian plants (14, 17 and 18), we have now evaluated the anti- malarial effect of different extracts of three *Scrophularia* species. The genus *Scrophularia* (Scrophulariaceae), comprising about 300 species, is one of the largest and most popular herbs in folk medicines, distributed in the northern hemisphere, especially along the Mediterranean area, central Europe, Asia and North America (19). In Iran, 42 species are reported, 19 of which are considered as endemic taxa (19). Several activities were reported by these plants such as anti-inflammatory, antibacterial, immunomodulator, cardiovascular, diuretic, molluscicidal, anti-tumor, and anti-malarial. Moreover, various types of phenolic acids, iridoids, phenylpropanoids, flavonoids, saponins, and terpenoids were isolated from this genus that may cause the mentioned pharmacological effects (20-29). The aim of the present study is to screen the anti-malarial activity of different extracts of three Irainan endemic species of *Scrophularia *including* S. frigida, S. subaphylla, *and* S. atropatana *as well as the report of the preliminary phytochemical screening of potent extracts.

## Experimental

*Chemicals*

Hematin porcine, chloroquine diphosphate, sodium dodecyl sulfate (SDS), sodium acetate, magnesium sulfate, sodium hydrogen phosphate, sodium chloride, potassium chloride, sodium hydroxide, glucose, and sodium bicarbonate were purchased from Sigma-Aldrich, chemical Company (United kingdom), oleic acid from Fluka (India), dimethylsulfoxide, hydrochloric acid. All the solvents used for extraction were purchased from Caledon (Canada).

*Plant Material*

The aerial parts of three *Scrophularia* species, *S. frigida*, *S. subaphylla* and *S. atropatana *were collected from East Azarbaijan province (Iran) during June-September 2012. After identification, voucher specimens numbered as Tbz-fPh-746, Tbz-fPh 747, and Tbz-fPh 748 respectively and also retained in the herbarium of the Faculty of Pharmacy, Tabriz University of Medical Sciences, Iran. 

*Extraction*

All of the samples were Soxhlet-extracted with n-hexane, dichloromethane (DCM), and methanol (MeOH), successively (1 L each). All the extracts were concentrated using a rotary evaporator under 45 ºC. 

*TLC analysis of extracts*

In the case of potent extract, important chemical groups were identified by TLC on silica gel 60 GF _254 _Merck (layer thickness 0.25 mm) as follows: n-hexane/ethylacetate (70:30) was used as solvent system and then detected under UV 366 nm.

*Cell free β-Hematin formation assay*

The potential anti-malarial activity of plant extracts was evaluated by the method described by Afshar *et al.* (17) with some modifications. Briefly, varying concentrations (0–2 mg/mL in DMSO) of the different extracts were incubated with 3 mM of hematin, 10 mM oleic acid, and 1 M HCl. The final volume was well adjusted to 1 mL by adding sodium acetate buffer, pH 5. Subsequently, overnight incubation at 37 °C consistent with shaking was considered for samples. During this process, chloroquine diphosphate was used as a positive control. Then, the samples were centrifuged (14000 rpm, 10 min, at 21 °C) and 2.5% (w/v) SDS in phosphate buffered saline frequently added to samples in order to purify the hemozoin pellets (usually 3-8 washes). This process was followed by a final wash in 0.1 M sodium bicarbonate until the supernatant was clear. Finally, the pure pellets were dissolved in 1 mL of NaOH and the absorbance was measured at 400 nm by UV spectrophotometer. The results were recorded as % inhibition (I%) of heme polymerization/crystallization compared to positive control (chloroquine) using the following formula: I% = [(AN – AA)/AN] × 100, where AN: absorbance of negative control; AA: absorbance of test samples.

*Preliminary Phytochemical Analysis*

The extracts were tested for identifying the active chemical groups such as triterpenoids, steroids, glycosides, saponins, alkaloids, flavonoids, tannins, free amino acids, and carbohydrate by the following standard procedures.

*Tests for steroids and triterpenoids*

Few drops of acetic anhydride were mixed with sulfuric acid which was added from the sides of the test tubes then brown ring was appeared at the junction of two layers which was surrounded with green layer at the top and deep red layer at the down. This test indicated the presence of steroids and triterpenoids respectively (30-32).

*Tests for cardiac glycosides*

A) Kedd ΄s test: The plant extracts were mixed with 2-3 drops of 2% 3, 5-dinitro benzene carboxylic acid. Then, 20% NaOH was added to make the solutions in alkali range. Finally, the appearance of purple color illustrated the presence of β-unsaturated lactones which gave positive response to the test (30).

B) Keller-killiani test: The mixture of glacial acetic acid and ferric chloride were added to dried test solutions. Detection of the color changed to bluish green in upper layer and reddish in down layer occurred after adding the concentration H_2_SO_4_, slowly by the side of the test tubes (30).

*Tests for alkaloids*

A) Dragendorff’s test: The development of reddish brown turbidity in the presence of Dragendorff reagent was indicative of the presence of alkaloids (30).

B) Hagerʹs test: 2-3 drops of Hager reagent were added to the extract tubes to observe yellow turbidity in the presence of alkaloids (30).

*Test for tannins and phenolic compounds:* Blue green color was appeared after adding Ferric chloride to the test solutions (30, 32). 

*Test for flavonoids (Shinoda test)*

After adding the mixture of Magnesium pieces and concentrated HCl to the samples, the red color was appeared (32).

*Test for amino acids (Ninhydrin test)*

The presence of free amino acids was resulted from the formation of purple color, when the solutions were boiled with 0.2% ninhydrin solution (30).

*Test for carbohydrate*

(Benedict’s test): The solution was treated with amount of Benedict’s reagent (alkaline solution containing cupric citrate complex), then boiling on water bath, reddish brown turbidity appeared if reducing sugars are present (30).

*Test for iridoids*

One mL of Trim-Hill reagent was added to the different extracts and then was heated for a few min. A blue-green or red color indicated the presence of iridoids (34).

*GC-MS Analysis of potent extract*

GC–MS analyses were carried out on a Shimadzu QP-5050A GC–MS system equipped with a DB-1 fused silica column (60 m × 0.25 mm i.d., film thickness 0.25 μm). For nonpolar extracts oven temperature, rising from 50 °C to 230 °C at a rate of 4 °C/min and then rising from 230 °C to 310 °C at a rate of 1.5 °C/min, injector temperature, 280 °C; carrier gas, helium at a flow rate of 1.3 mL/min; split ratio, 1:10; ionization energy, 70 eV; scan time, 1 sec; mass range, 30–600 amu.

*Identification of components*

Identification of the constituents was based on direct comparison of the retention times and mass spectral data with those for standard alkanes and computer matching with the NIST 21, NIST 107 and WILEY229 library, as well as by comparison of the fragmentation patterns of the mass spectra with those reported in the literature (35).

*Statistical analysis*

All measurements were expressed as the Mean ± SD in triplicate manner. Excel 2010 was employed for analyzing data. The IC_50_ value was calculated from nonlinear regression analysis.

## Results

*Cell free β-hematin formation assay results*

**Figure 2:**
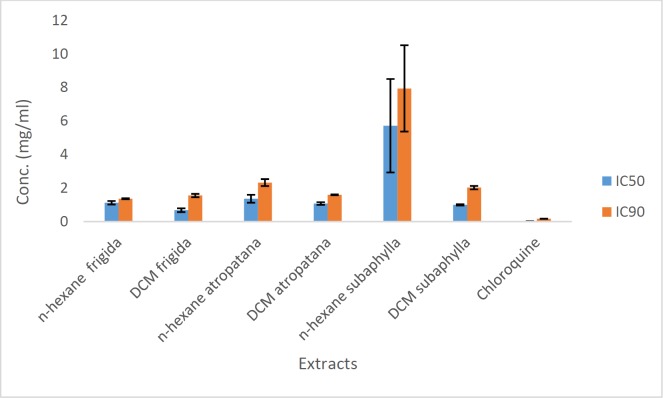
Comparison of IC_50_ and IC_90_ values (mg/mL) of active extracts of *S. frigida*, *S. subuphylla*, *S. atropatana* and chloroquine solution in β-hematin formation assay. The values were reported as Mean ± SD

The results of cell free β-hematin formation assay which was carried out on three different extracts of three *Scrophularia* species were compiled in Table 1 and Figure 2*. *MeOH extracts of all specimens including *S. frigida, S. subaphylla* and* S. atropatana* showed no anti-malarial activity, while all DCM extracts exhibited potent anti-malarial effect with IC_50 _values of 0.67 ± 0.11, 0.99 ± 0.04 and 1.07 ± 0.07 mg/mL, respectively, in comparison to positive control (chloroquine, IC_50 _= 0.014 ± 0.003 mg/mL). Moreover, medium potencies were illustrated by n-hexane extracts with IC_50_ values of 1.12 ± 0.10, 5.74 ± 2.80, and 1.35 ± 0.20 mg/mL), respectively. Amongst the extracts, DCM extract of *S. frigida* illustrated the most potent anti-malarial activity.

**Table 1.** The 50% and 90% of inhibition concentrations (mg/mL) of extracts of three *Scrophularia *species in 𝛽-hematin formation assay

**Plants**	**Extracts**	**Yield (%)**	**IC**_50 _ **(mg/mL)**	**IC**_90 _ **(mg/mL)**
*Scrophularia frigida*	n-hexane	3.6	1.12 ± 0.10	1.36 ± 0.028
	DCM	3.52	0.67 ± 0.11	1.55 ± 0.100
	MeOH	12.13	-	-
*Scrophularia subaphylla*	n-hexane	3.49	5.74 ± 2.81	7.94 ± 2.57
	DCM	5.56	0.99 ± 0.04	2.02 ± 0.105
	MeOH	13.24	-	-
*Scrophularia atropatana*	n-hexane	2.24	1.35 ± 0.20	2.32 ± 0.21
	DCM	6.32	1.07 ± 0.07	1.59 ± 0.021
	MeOH	14.23	-	-
Chloroquine	-	-	0.014 ± 0.003	0.163 ± 0.004

Experiment was performed in triplicate and expressed as Mean ± SD.

**Table 2:** Phytochemical characteristics for various extracts of three different species of *Scrophularia*

**Phytochemical tests**		**n-hexane extract**			**DCM extract**			**MeOH extract**		
		***S. f***	***S. s***	***S. a***	***S. f***	***S. s***	***S. a***	***S. f***	***S. s***	***S. a***
Alkaloids	Dragendorff’s Test	-	-	-	-	*-*	-	+	-	-
	Hager’s Test	-	-	-	-	-	-	+	-	-
Cardiac Glycosides	Kedd	+++	+++	+++	+++	+++	+++	-	-	-
	Keller-killiani	-	+	+	-	+++	-	-	-	
Tannins	Ferric Chloride Test	-	-	-	-	-	-	+++	+++	+++
Flavonoids	Shinoda Test	-	-	-	++	-	-	+++	+++	+++
Proteins and Amino Acids	Ninhydrin Test	-	-	-	-	-	-	-	-	-
Sterol	Libermann-Buchard test	+++	+	+	-	+++	+	-	-	-
Terpenoid	Libermann-Buchard test	-	-	-	+++	++	+	-	-	-
Carbohydrate	Benedict's test	-	-	-	-	-	-	+	+	+
Iridoids	Trim-Hill	-	-	-	-	-	-	+	+	+

* S. f* = *S. frigida; S. s = S. subaphylla; S. a = S. atropatana; *MeOH = methanol*; *DCM = dichloromethane.

**Table 3:** Volatile components of DCM and n-hexane extracts in three species of *Scrophularia*

**Extracts**	**Total identified content (%)**	**Compound (content %)**
**DCM**		
*S. atropatana*	95.33	*n*-alkanes (84.92%), diterpene (1.3%), fatty acids and their derivatives (8.17%), heterocyclic hydrocarbons (0.94%)
*S. frigida*	95.95	diterpene (69.02%), fatty acid (26.91%)
*S. subaphylla*	74.65	diterpene (59.87%), linear alcohol (9.33%), fatty acid (5.45%)
**n-hexane**		
*S. atropatana*	95.04%	oxygenated monoterpens (14.76%), fatty acid (2.42%), alkanes (77.86%)
*S. frigida*	96.13%	Fatty acids and their derivatives (46.96%), alkanes (39.73%), linear ketone (1.22%), steroids (8.22%)
*S. subaphylla*	80.09%	fatty acid (1.83 %), alkanes (78.26%)

*Phytochemical analysis results*

In order to confirm our antimalarial results, preliminary phytochemical analysis, TLC, and GC-MS analysis were performed. Based on phytochemical analysis data (Table 2), high amounts of flavonoids and terpenoids in DCM extract (as a potent extract) and steroids in n-hexane extract (as a medium potent extract) of *S. frigida *were demonstrated. Furthermore, the presence of cardiac glycosides with their steroidal moiety might be effective in the potency of DCM and n-hexane extracts. In addition, it is assumed that the flavonoids and coumarins which existed in DCM extract may be lipophilic types (for instance, methoxylated or methylated things). Furthermore, according to Table 3, GC-MS analysis of volatile parts of DCM and n-hexane extracts showed high amounts of diterpenoids and steroids in *S. frigida*, respectively.

## Discussion

The *Plasmodium* parasite attacks the host erythrocyte in order to utilize hemoglobin for synthesizing the essential requirements for developing and proliferating (36, 37). During this process, a massive amount of heme is generated as a toxic undesired byproduct which is pernicious for malaria parasite. Therefore, to protect itself, the parasite neutralizes large amounts of heme to hemozoin or water-insoluble malaria pigment via the bio crystallization process. Hence, inhibition of hemozoin formation by means of antimalarial compounds like artemisinin and 4-aminoquinolines derivatives (quinine, mefloquine and chloroquine) is regarded as an incomparable target to combat the malaria (14, 38). Despite of the available drugs, researchers have decided to screen the plants for discovering the novel natural sources especially from endemic flora (39). Hence, in this current study, three endemic species of *Scrophularia *were selected for evaluating their anti-malarial activities. Obtained results revealed that the MeOH extracts of all plants did not show any significant anti-malarial activities whereas the DCM and n-hexane extracts of all aerial parts indicated high to moderate anti-malarial potency in comparison with the reference control. Amongst them, DCM extract of *S. frigida* showed remarkable anti-malarial activity. It seems that potent effect of DCM extract from *S. frigida* on inhibiting the hemozoin formation was may be due to the presence of high amounts of flavonoids and terpenoids as resulted from the preliminary phytochemical analysis (Table 2). Moreover, TLC analysis indicated the presence of methylated coumarins and methoxylated flavonoids in DCM extract of* S. frigida* in comparison with the reference book (40). Our findings are parallel with the prior investigations which exhibited anti-plasmodial activity of methoxylated flavonoids (41), methylated coumarins (42) and terpenes (43-46) in various anti-malarial assays. Furthermore, in previous studies on *Scrophularia* genus, antimalarial activity of some species was demonstrated (23, 28). In addition, GC-MS analysis of DCM extracts of three species were performed for determining the probable anti-malarial volatile compounds (Table 3). In the case of *S. atropatana*, *n*-alkanes comprised the highest proportion while in two other species diterpenes were identified as the major constituents. Hence, it could be supposed that diterpenes as the major active compounds are responsible for the potent activity of those extracts (43). Additionally, among the n-hexane extracts, the anti-malarial effect of n-hexane extract of *S. frigida* was found to be more potent than the others. Likewise, although the preliminary phytochemical screening indicated the presence of steroid derivatives in all n-hexane extracts, (Table 2), a considerable quantity of steroids in n-hexane extract of *S. frigida* could explain the potent activity of this extract. Furthermore, GC-MS analysis of this extract was in line with the findings of phytochemical analysis (Table 3). 

However, IC_50 _value of this extract was higher than negative control which might be due to the existence of high amounts of fatty acids and lipids leads to synergistic effect with oleic acid and conceal the authentic anti-malarial potency of the extract in this assay. Hence, antimalarial activity of this extract could be increased by removing fatty acids (14). Likewise, anti-malarial activity of steroids was illustrated previously in other plants (43, 44). In the case of n-hexane extract of *S. atropatana*, moderate antimalarial effect in comparison to others and positive control might be due to the presence of oxygenated monoterpenes in volatile part (43). 

## Conclusion

Based on this preliminary study, among the various polarity extracts of three different plants, DCM extract of *S. frigida* showed the significant anti-malarial activity on β-hematin formation assay. These results encouraged us to concentrate on isolating the active anti-malarial chemical constituents and studying further on animal models for *in-vivo* evaluation.
